# Effect of Laser Pulse Overlap and Scanning Line Overlap on Femtosecond Laser-Structured Ti6Al4V Surfaces

**DOI:** 10.3390/ma13040969

**Published:** 2020-02-21

**Authors:** Georg Schnell, Ulrike Duenow, Hermann Seitz

**Affiliations:** 1 Microfluidics, Faculty of Mechanical Engineering and Marine Technology, University of Rostock, Justus-von-Liebig-Weg 6, 18059 Rostock, Germany; ulrike.duenow@uni-rostock.de (U.D.); hermann.seitz@uni-rostock.de (H.S.); 2 Department Life, Light & Matter, University of Rostock, Albert-Einstein-Str. 25, 18059 Rostock, Germany

**Keywords:** femtosecond laser, ultrashort laser pulse, ablation threshold, Ti6Al4V, laser pulse overlap, scanning line overlap

## Abstract

Surface structuring is a key factor for the tailoring of proper cell attachment and the improvement of the bone-implant interface anchorage. Femtosecond laser machining is especially suited to the structuring of implants due to the possibility of creating surfaces with a wide variety of nano- and microstructures. To achieve a desired surface topography, different laser structuring parameters can be adjusted. The scanning strategy, or rather the laser pulse overlap and scanning line overlap, affect the surface topography in an essential way, which is demonstrated in this study. Ti6Al4V samples were structured using a 300 fs laser source with a wavelength of 1030 nm. Laser pulse overlap and scanning line overlap were varied between 40% and 90% over a wide range of fluences (*F* from 0.49 to 12.28 J/cm²), respectively. Four different main types of surface structures were obtained depending on the applied laser parameters: femtosecond laser-induced periodic surface structures (FLIPSS), micrometric ripples (MR), micro-craters, and pillared microstructures. It could also be demonstrated that the exceedance of the strong ablation threshold of Ti6Al4V strongly depends on the scanning strategy. The formation of microstructures can be achieved at lower levels of laser pulse overlap compared to the corresponding value of scanning line overlap due to higher heat accumulation in the irradiated area during laser machining.

## 1. Introduction

The topography of implants affects the cellular response and, consequently, the implant performance [1,2,3,4,5,6]. Therefore, the surfaces of titanium, as a widely common material for many biomedical applications, such as dental and orthopedic implants, has been treated in various ways to adjust a proper surface design. Sandblasting [7,8,9,10,11], chemical etching [9,10,11], and coatings [10] have been used for roughness modification of titanium surfaces for a long time and have been well investigated. Laser treatment of titanium has been found to be particularly advantageous compared to conventional surface structuring methods because it leads to less contamination of the surfaces [12] and offers the possibility of creating stochastic as well as precise deterministic structures in the micro- and nanometer range [5,13,14]. In particular, the potential of femtosecond (fs) laser structuring of titanium for use in biomedical implants has been shown in several studies [15,16,17,18]. This technique is superior to nanosecond laser treatments due to opportunity of creating surface topographies with a greater variety of patterns [19]. Basically, ultra-short pulse laser machining, such as fs laser structuring, also offers many advantages over short pulse laser treatment [20,21], for instance, a widely reduced deposition of debris and trace of melting or molten material [19].

Several studies focused on the effect of different fs laser parameters on the formation of nano- and microstructures on titanium surfaces. For example, the influence of laser fluence as well as the number and duration of pulses on the formation of surface structures on titanium has been studied. It has been demonstrated that the spatial periodicity of ripples is affected by the laser pulse duration, which ranges from 200 fs to 800 fs [16]. Furthermore, an increasing number of pulses per spot leads to a decreasing spatial periodicity of ripples [16], whereas the spatial periodicity of microstructures rises with a growing number of laser pulses [22]. An increase of the spatial periodicity of micrometric ripples (MR) has been observed with a rising number of laser pulses on Ti6Al4V surfaces [23]. Moreover, the formation of sharp conical microstructures on titanium surfaces due to fs laser irradiation in vacuum and in a helium atmosphere has been shown [24]. Other studies investigated the formation of highly regular laser-induced periodic surface structures [25] and the manipulation of the nanotopology by a spatially asymmetric fluence on titanium surfaces leading to the result that a shaped laser beam irradiation can result in smooth microstructures in contrast to a laser treatment with a Gaussian beam profile, which typically results in a pronounced double-scale roughness [26].

The process parameters laser pulse overlap and scanning line overlap, which also strongly affect the formation of structures on Ti6Al4V, have not been examined thoroughly in previous studies. This study systematically investigates the effects of both laser parameters on the surface texture of Ti6Al4V at different fluences. The laser pulse overlap is varied whereas the scanning line overlap is maintained constant and vice versa. Essentially, the resulting different dynamics of the fs laser structuring process are analyzed. The results can ultimately be used to optimize the scanning strategies for fs laser surface machining.

## 2. Materials and Methods

### 2.1. Material

The experimental investigations were carried out with Ti6Al4V samples that were cut by waterjet technology to a plate size of 8 cm × 6 cm × 0.4 cm. The plates were purchased from S + D Spezialstahl Handelsgesellschaft mbH, Stelle, Germany and the specifications of the material complies with the requirements of AMS4911 and WL 3.7164 Part 1. In order to ensure uniform surfaces with low roughness, the samples were polished with silicon carbide abrasive sandpaper under pure water in three steps. Initially, sandpaper with a grain size of P320 was applied for 8 min followed by sandpaper with a grain size of P600 for 4 min and then a grain size of P1200 for a further 4 min resulting in an average area surface roughness *Sa* of approximately 0.065 ± 0.003 µm. The abrasive treatment was followed by cleaning in a Sonorex Super RK 100/K ultrasonic bath (Bandelin electronic GmbH and Co. KG, Berlin, Germany) with ultrapure water for 15 min. Drying was applied with dust-free wipes and compressed air.

### 2.2. Laser Treatment

**Figure 1:**
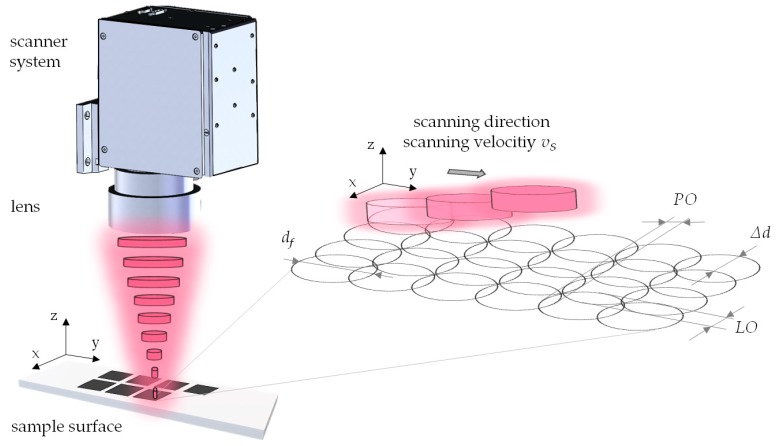
Schematic illustration of the laser surface structuring using a line-wise scanning strategy leading to a defined laser pulse overlap (*PO*) and scanning line overlap (*LO*). Deflecting in the x and y directions of the Gaussian laser beam is evoked by the movement of galvanic mirrors within the scanning system.

A 300 fs UFFL_60_200_1030_SHG fiber laser (Active Fiber Systems GmbH, Jena, Germany) featuring an amorphous glass Yb-doped core was used for laser structuring. The laser system is integrated into a Microgantry GU4 five-axis micromachining center (Kugler GmbH, Salem, Germany). The pulse repetition rate of the laser system can be varied from 50.3 kHz to 18.6 MHz, with an average power of up to 60 W. The laser emits linear polarized light with a wavelength of 1030 nm. An f-theta lens with a focal length of 163 mm leads to a circular focus diameter of *d_f_* = 36 microns at 1/e² intensity (Gaussian laser beam profile). This spot diameter is used for all laser parameter calculations. A schematic illustration of the scanning arrangement and related scanning parameters are given in Figure 1.

The laser pulse overlap *PO* is calculated from Equation (1):

(1) $$PO=\left(1-\frac{v_{S}}{d_{f}\;\times \;f_{REP}}\right)\times \;100\%$$

where *v_S_* is the scanning velocity, *d_f_* the circular focus diameter and *f_REP_* the repetition rate. The *LO* is calculated according to Equation (2):

(2) $$LO=\left(1-\frac{\Delta d}{d_{f}}\right)\times \;100\%$$

where Δ*d* is the space in scanning line arrangement and *d_f_* denotes the focus diameter. The *PO* is varied in a range from 40% to 90% at a fixed *LO* of 50% and vice versa. It is reported that the forming of periodic ripples on titanium surfaces starts at a fluence *F* of about 0.5 J/cm² [27]. Accordingly, this value is used as the starting point for the applied fluence. Fluence is increased systematically, aiming at extensive formation of microstructures. Consequently, for every combination of *PO* and *LO*, the pulse energy is varied from 5 to 125 µJ leading to a corresponding fluence range from 0.49 to 12.28 J/cm^2^ (see Table 1). In the following, special attention is given to the fluence as the crucial laser parameter in material processing.

The repetition rate of the laser was maintained constant at *f_REP_* = 226.8 kHz to ensure a constant time interval between consecutive laser pulses. The different *POs* are achieved by the adjustment of the scanning velocities (*v_S_* from 0.82 to 4.9 m/s) in accordance with Equation (1) and different *LOs* are realized by setting corresponding spacings (Δ*d* from 4 to 32 µm) between the scanning lines in accordance with Equation (2). The number of overscans was maintained at a constant of 50. The structuring area is 0.7 cm × 0.7 cm.

### 2.3. Surface Characterisation

A LEXT OLS 4000 confocal laser scanning microscope (CLSM) (Olympus, Hamburg, Germany) was utilized to determine the elevation profile (depth and width of the resulting pillars and profiles, respectively) and the average area surface roughness (*Sa*). A constant optical magnification (50×) was used leading to a scan area of 256 µm × 256 µm. The resulting scans have a resolution of 1024 × 1024 pixels. For data calculation and visualization, OLS4000 software (Version 2.2.3, Olympus, Hamburg, Germany) was applied. For detailed images, a StereoScan360 scanning electron microscope (SEM) (Cambridge Instruments, Cambridge, UK) was used. The spatial periodicities of the femtosecond laser-induced periodic surface structures (FLIPSS) *λ_1_* and of the micrometric ripples (MR) *λ_2_* were determined by measuring the distances between periodic structures in the SEM images using ImageJ software (Version 1.52, available as freeware online from https://imagej.nih.gov/ij/), respectively. Each measurement was repeated five times.

## 3. Results and Discussion

### 3.1. Surface Characterization

**Figure 2:**
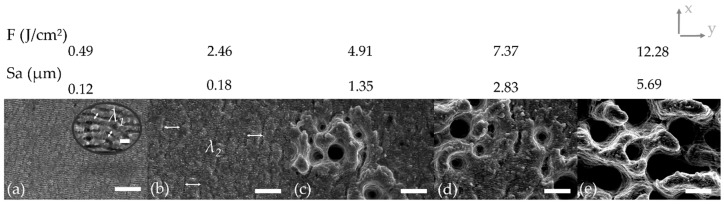
Formation of nano- and microstructures at a *PO* of 50% and *LO* of 80% with increasing laser fluence *F*. The y-axis indicates the scanning direction for the *PO.* The x-axis indicates the line feed direction for the *LO*. (**a**) Femtosecond laser-induced periodic surface structures, *λ_1_* = 0.859 ± 0.056 µm, scale bar 10 µm and in detail 1 µm. (**b**) Micrometric ripples, *λ_2_* = 5.038 ± 0.829 µm, scale bar 10 µm. (**c**,**d**) Formation of micro-craters, scale bar 10 µm. (**e**) Pillared microstructures, scale bar 10 µm.

All structuring experiments have been performed successfully leading to surfaces with different structural characteristics depending on the applied laser parameters and scanning strategy. When considering the results, four different types of structures can be basically distinguished. Figure 2 exemplarily shows the resulting structures for the structuring parameters *PO* of 50% and *LO* of 80% for different fluences. Structures can be found on the nanometer-scale, so-called femtosecond laser-induced periodic surface structures (FLIPSS) featuring a spatial periodicity *λ_1_* (see Figure 2a), as well as on the micrometer-scale, where micrometric ripples (MR) with a spatial periodicity *λ_2_* (see Figure 2b) can be distinguished from micro-craters (see Figure 2c,d) and pillared microstructures (see Figure 2e). Microstructures are typically superimposed by nanostructures.

The ablation threshold, which represents the minimum energy density to remove material by laser treatment, plays a key role when considering the structuring results. The ablation threshold principally depends on the applied laser pulse duration [28], the laser wavelength [29], the number of pulses applied on the irradiated spot [30,31] and on the material itself [32]. Fluences slightly higher than the ablation threshold lead to the formation of FLIPSS. Interferences between the linearly polarized laser light and the excited surface plasmon polaritons lead to a uniform formation and orientation of these structures. The periodic FLIPSS arranged perpendicularly to the laser electric field polarization vector and the periodicity match the wavelength or is slightly smaller than the applied wavelength [22,31,33,34]. The determined spatial periodicity of the FLIPSS in Figure 2a in the study is *λ_1_* = 0.859 ± 0.056 µm and, therefore, slightly lower than the applied laser wavelength of 1030 nm. Furthermore, micro-ripples can be observed that are oriented parallel to the polarization of the laser beam. The formation of MR is related to hydrodynamic effects due to heat exposure in the liquid area by melting processes [23]. This double-scale formation of structures is caused by nonuniformity of the intensity distribution of the Gaussian laser beam and uniform motion of the spot, which is evoked by deflecting the laser light by the scanner system in this study [35]. The spatial periodicity of the MR in Figure 2b was determined as *λ_2_* = 5.038 ± 0.829 µm. Low laser fluences in this so-called gentle ablation lead to an increase in temperature on the surface and, consequently, to melting and low evaporation, or rather sublimation of metals in tens of nanometers [36]. Therefore, the amount of ablated material depends on the optical penetration depth [31,37,38]. Melt splashing in this regime is negligible and phase explosion did not occur [32]. Once the fluence exceeds the strong ablation threshold, the ablation rate depends on the electron heat diffusion length [31,37] and is determined by the effective heat penetration depth [38]. The heat penetration depth characterizes the distance of effective heat energy transport via electrons [31]. The change of the ablation regime is explained by non-linear heating (superheating) of electrons in the conduction band [36,38] and the ablation in the regime is caused by phase explosion [31,36,39]. Particles mixed with the overheated liquid phase are emitted [30] and the resulting surface is substantially coarser leading to an increased roughness, as shown in Figure 2c–e. Phase explosion, also referred to as explosive boiling, results in an immense ejection of vapor and molten material that leads to a rapid and very large increase of the ablation rate [32,39] which can be observed in Figure 2e at a fluence of 12,28 J/cm². A more precise gradation of the ablation regimes is given in [32] where melt displacement and melt splashing are considered as new regimes between gentle and strong ablation. Melt displacement and melt splashing are explained by internal inhomogeneous vaporization of matter with different elements or external effects of vapor recoil or generated plasma pressure [32]. Melt displacement can be observed in Figure 2c,d for the used titanium alloy and starts at a fluence of 4.91 J/cm² at a *PO* of 50% and *LO* of 80%. Furthermore, the Gaussian-shaped laser beam features fluences in the strong ablation regime in the center of the spot whereas the intensity of the laser beam in the surrounding area is much lower. This leads to a partial coverage of the microstructures with nanostructures.

### 3.2. Effects of Laser Pulse Overlap and Scanning Line Overlap on Texturing of Ti6Al4V

**Figure 3:**
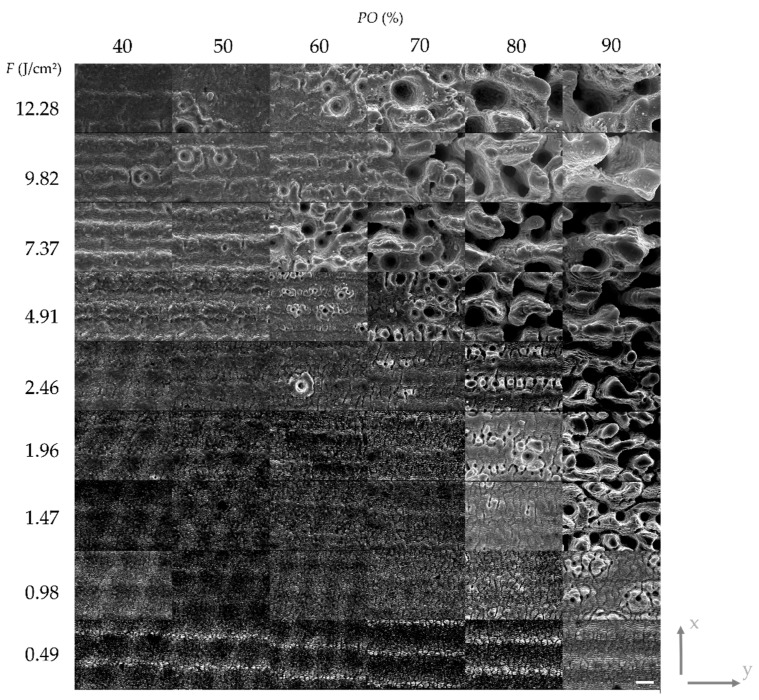
Structured Ti6Al4V surfaces with varied fluence *F* and laser pulse overlap *PO* in a matrix of SEM images. The y-axis indicates the scanning direction for the *PO.* The x-axis indicates the line feed direction for the *LO*. Formation of microstructures can be observed at a fluence of approx. 0.98 J/cm² and higher. In particular at low fluences, a clear formation of trenches can be observed due to the *LO* of 50% and inhomogeneous energy distributions within the Gaussian laser beam profile. By comparison of the structures irradiated with a constant *PO* of, e.g., 90%, it can be observed that the ablated craters become larger in terms of diameter and depth with increasing fluence (compare roughness data in Figure 7 and elevation profile in Figure 5). Scale bar: 10 µm.

**Figure 4:**
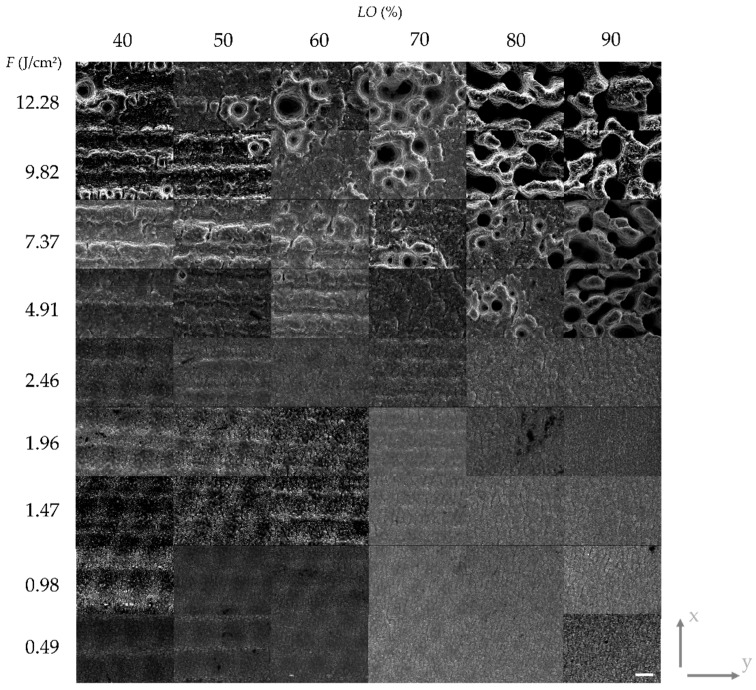
Structured Ti6Al4V surfaces with varied fluence *F* and scanning line overlap *LO* in a matrix of SEM images. The y-axis indicates the scanning direction for the *PO.* The x-axis indicates the line feed direction for the *LO*. Formation of microstructures can be observed at a *F* of approx. 4.91 J/cm² and higher. With an increasing *LO*, a more homogeneous surface with smoother trench formation is apparent (confirmed by elevation height profiles in Figure 6). Scale bar: 10 µm.

**Figure 5:**
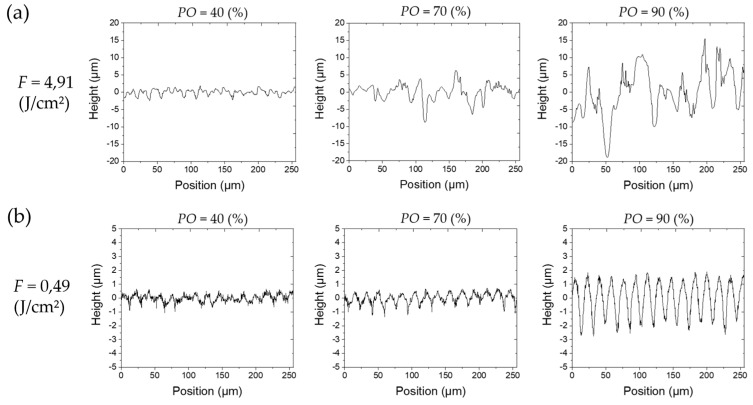
Exemplary height elevation profiles in x-direction for different *PO* (40%, 70%, and 90%) and different fluences (*F* = 4.91 J/cm² and *F* = 0.49 J/cm²), respectively. (**a**) A clear formation of stochastic microstructures can be observed at a high level of *PO* at *F* = 4.91 J/cm². Width and height of the structures rise with increasing *PO*. (**b**) At *F* = 0.49 J/cm², the height of deterministic structures grows and periodicity remains constant with increasing *PO*. Structures are covered with nanoscale roughness.

**Figure 6:**
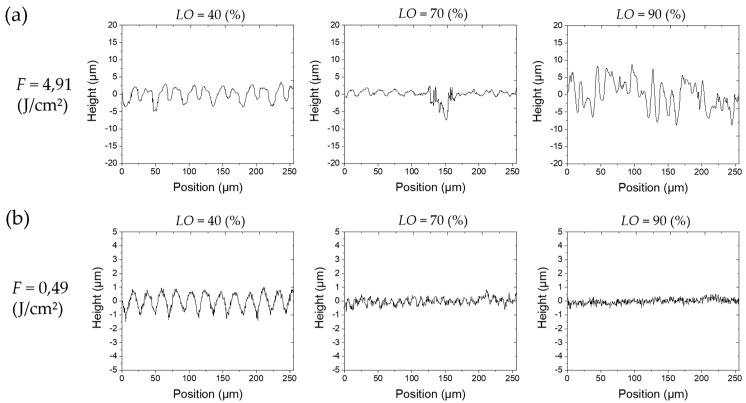
Exemplary height elevation profiles in x-direction at different *LO* (40%, 70% and 90%) and different fluences (*F* = 4.91 J/cm² and *F* = 0.49 J/cm²). (**a**) Low levels of *LO* lead to considerable formation of trenches (*LO* = 40%). Firstly, structures become smaller with increasing *LO* (*LO* from 40% to 70%). After reaching the high ablation regime, stochastic microstructures gradually grow with increasing *LO* at high levels of *F.* (**b**) The periodicity and height of structures decreases with increasing *LO*. High *LO* leads to a clear homogenous nanoscale roughness.

The results of the parameter study with variable *PO* at a fix *LO* of 50% are shown in Figure 3 and with variable *LO* at a fix *PO* of 50% in Figure 4, respectively. The results are depicted as a matrix of SEM images where the rows correspond to certain levels of fluence. The presented results at a certain position in matrices in both figures have been laser-treated with the same total energy since the levels of *PO* and *LO*, respectively, are identical. Furthermore, the y-axis indicates the scanning direction for the laser beam (and, consequently, the direction for the *PO*) whereas the x-axis indicates the line feed direction for the *LO* (Figure 3 and Figure 4). Figure 5 and Figure 6 show height elevation profiles for the structures created at two selected levels of fluence (*F* = 4.91 J/cm² and *F* = 0.49 J/cm²) for three levels of *POs* (40%, 70%, and 90%) and for three levels of *LOs* (40%, 70%, and 90%), respectively. The associated SEM images of the structures can be found at the corresponding positions in the matrices in Figure 3 and Figure 4, respectively.

**Figure 7:**
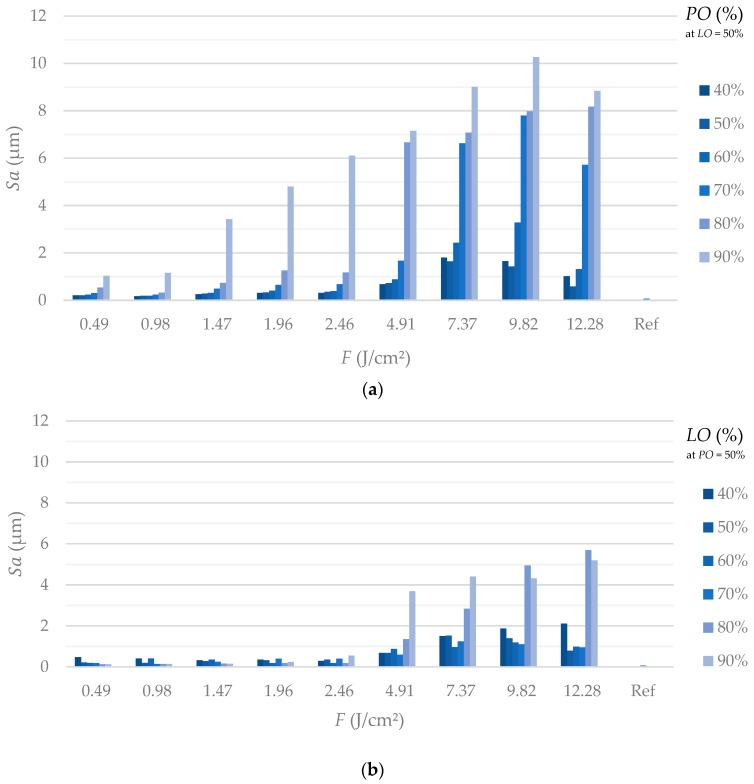
Average area surface roughness (*Sa*) after laser processing related to the reference (Ref). (**a**) A clear increase of the roughness can be obtained with increasing *PO* at all fluences. (**b**) A higher scanning line overlap leads also to an increase in roughness above a fluence of approx. 4.91 J/cm². Firstly, a higher *LO* leads to a decreased roughness at low fluences. Generally, the determined average area surface roughness is less compared to that obtained at corresponding levels of *PO* (and the same energy input). At fluences from 7.37 J/cm² to 12.28 J/cm², a minor increase in roughness can be detected at a low level of *LO* due to a clear trench formation.

Through comparison of Figure 3 and Figure 4 it can be concluded that the different scanning strategies have a major impact on the formed structures. Firstly, a low level of *LO* leads to a clear formation of trenches in scanning direction. This effect can clearly be seen in Figure 3 at low fluences. Figure 5b also shows that the depth of the trenches increases with increasing fluence. A low level of *PO* results in the formation of dimple-like cavities, especially at low fluences as depicted in Figure 3. On the one hand, trenches can be avoided if the fluence exceeds the strong ablation threshold and microstructures are formed, as shown in Figure 5a. On the other hand, trenches can also be avoided by a sufficient level of *LO*, as can be seen in Figure 4 and Figure 6. In particular, a sufficient level of *LO* leads to a homogenous formation of nanostructures, as depicted in Figure 6b. The roughness data in Figure 7b reflects the decrease in waviness by a lower *Sa* with increased levels of *LO*. Apart from fluences higher than the ablation threshold, all effects can be attributed to inhomogeneous energy distributions within the Gaussian laser beam profile and, therefore, nonuniform ablation. At a certain level of superimposition of laser pulses, a widely homogenous surface can be achieved.

It can be deduced from Figure 3 and Figure 7a, that high levels of *PO* lead to a formation of microstructures already at low fluences. In contrast, Figure 5 and Figure 7b demonstrate that, at the corresponding levels of *LO*, microstructures only formed at higher fluences (e.g., compare *PO* of 90% at 1.47 J/cm² in Figure 3 and *LO* of 90% at 1.47 J/cm² in Figure 4). Even if the applied fluence is the same, it seems that the time sequence of the series of pulses plays a major role, especially in achieving the strong ablation threshold. This phenomenon is also confirmed by the comparison of roughness values resulting from different *POs* and *LOs*, as seen in Figure 7. Basically, the average area surface roughness *Sa* increases more at certain high levels of *PO* than at corresponding levels of *LO*. Similar values for the *Sa* only achieved at higher fluences for the *LO* if comparing corresponding levels of *PO* and *LO* (e.g., *Sa* = 3.42 µm for *PO* = 90% at *F* = 1.47 J/cm² vs. *Sa* = 3.684 µm for *LO* = 90% at *F* = 2.46 J/cm²). To gain a better understanding of the responsible reasons for differences in *PO* and *LO* texturing, it is necessary to consider the dynamic of the ablation process in detail at various time-scales.

When discussing the creation of the surface structure depending on the different *LO* and *PO* parameters, the dynamics of the laser structuring process itself has to be considered. Firstly, when looking at a single femtosecond laser pulse, heat conduction occurs if the boiling temperature is not exceeded [40]. Initial absorption of the laser irradiation of a laser pulse occurs from the free electrons in the first femtoseconds due to inverse bremsstrahlung. Subsequently, fast energy relaxation in the electron subsystem, energy transfer to the lattice (induced by electron-phonon coupling) and thermal diffusion in the material occurs [38,41]. If the material temperature crosses the boiling point of the material, evaporation or rather sublimation of material takes place as a direct solid-vapor/plasma transition [20]. This effect is caused by inhibited or restricted energy transverse of heated electrons to the lattice and rapid electron cooling at a time scale of around 1–3 ps [42,43,44]. Energy from the electron gas cannot be migrated into the ion lattice and leads to a temperature gap between electron and lattice [45]. Hot excited electrons are in a thermal non-equilibrium with high pressure in the first picoseconds [46]. Ultra-high heating rates lead to a transition to an overcritical fluid with a temperature close to the critical thermodynamic temperature [47] and thermionic and photoelectric effects lead to an emission of electrons [48]. This high-pressure mixture expands rapidly and leads to phase explosion [45]. A plasma plume is consequently formed afterwards with constituents of electrons in tens of picoseconds. Atomic, or rather ionic, mass is ejected up to nanoseconds and greater particles (nm) are cast out in a microsecond time scale [49].

When considering multiple laser shots at high repetition rates, several effects occur. If the ablated material results in a plasma plume, interaction with the incoming laser light by the next laser pulse takes place and leads to an inefficiency of energy deposition in the laser spot, which is referred to as plasma shielding [40,49]. Photoionization of excited atoms and electron-ion as well as electron-neutral bremsstrahlung are the mechanisms that lead to an absorption of a part of the incident photons before hitting the target and, consequently, to an increase of the plasma temperature [32]. The absorption coefficient of the plasma is proportional to the amount of vaporized material [50,51]. Conglomerated material after laser ablation in air represent a further source for absorption of the incident light and is expected at a repetition rate of hundreds of kHz [41]. This study was conducted with a repetition rate of 226.8 kHz. Consequently, plasma shielding must be considered, especially at a high *PO*. However, even though the applied repetition rate was the same for *PO* and *LO*, plasma shielding cannot be the driving force for different surface characteristics since it would instead lead to a decrease in ablation at increasing levels of *PO* or *LO*.

Ablation threshold reduction by a sequence of laser pulses with low repetition rates is explained by chemical changes and crystallographic material modifications and shown on titanium nitride films [52], where heat dissipation has taken place and the initial surface temperature is given. Consequently, even a single laser pulse below the gentle threshold can lead to structural or chemical modifications [30]. For multiple laser pulse irradiation with low repetition rates, it was reported that previous pulses result in an accumulation of defects, specified as long-lived bulk defects, like Frenkel defects. The accumulation is related to a storage cycle of thermal stress-strain energy due to the number of pulses and explained by the incubation model [46]. Due to the accumulation of defects, phase explosion can be evoked at lower fluences after a sufficient number of incubation pulses at low repetition rates [36]. A reduction in the ablation threshold with an increasing number of laser pulses at stationary irradiation and a repetition rate of 100 Hz was shown on different metals and explained by the incubation effect [23]. Physical mechanisms of the incubation effect have not yet been fully elucidated. A strengthened incubation effect was observed for a repetition rate of over 600 kHz and can be explained by heat accumulation [53]. In this study, the repetition rate and number of over scans were maintained at a constant of 226.8 kHz and a number of 50, respectively, for all *PO* and *LO* parameters. Thus, the incubation effect due to structural changes must be taken into consideration, but the time sequence of the pulses and scanning strategy probably has the dominant role.

If a high repetition rate is used, yielded energy cannot dissipate. The following laser pulses lead to heat accumulation and heating of the illuminated area takes place. It is obvious, by considering Figure 3 and Figure 4, that the formation of microstructures and higher amount of ablation is achieved at lower levels of *PO* compared to *LO* at a constant energy input. This effect is confirmed by the height elevation profiles at high fluences, as depicted in Figure 5a and Figure 6a. Height profiles show, that microstructures are formed at higher levels of *LO* in comparison to corresponding levels of *PO*. Furthermore, a higher *PO* ablates more material even at lower fluences which ultimately results in higher roughnesses, as can be seen in Figure 7. The duration for the structuring of one line (7 mm) in this study is in the range of several milliseconds (*v_S_* from 0.82 to 4.9 m/s). The time interval between two consecutive laser pulses is several microseconds due to the repetition rate of 226.8 kHz. Thus, a higher *PO* seems to lead to a stronger accumulation effect than is the case at corresponding levels of *LO* due to a shorter time sequence in laser pulse irradiation. Heat dissipation through thermal conduction is insufficient and the temperature of the material in the irradiated spot gradually increases due to consecutive laser pulses applied on the same area. Consequently, pronounced phase explosion is evoked due to a higher *PO* and a formation of microstructures occurs at lower *PO* values compared to the corresponding *LO* values. Higher repetition rates usually result in lower pulse energies, which makes this conclusion interesting for multiple-beam laser treatment. By means of a nanosecond laser, an increase of crater depth was caused by the heat accumulation effect at repetition rates around 100–1000 Hz [54]. Consecutive laser pulses lead to a continuous heating of the illuminated area even at kilohertz repetition rates [30]. Consequently, the ablation process takes place at different surface temperatures. In an extreme case at a repetition rate of 133 MHz, quasi-CW heating effects have been shown on aluminum foils with a burst of mode-locked picosecond laser [50,55]. Therefore, with a repetition rate of 226.8 kHz applied in this study, it can be concluded that the aforementioned phenomena for high repetition rates are responsible for presented different surface characteristics achieved at corresponding levels of *PO* and *LO*. At high *POs*, microstructures can be formed at lower fluences and a higher roughness modification is evoked compared to laser structuring at corresponding *LOs*. Numerical models have shown the effect of heat accumulation on surface temperature in multi-pulse laser processing [56,57]. Adsorbed energy that is not used for rapid evaporation or sublimation of the material remains in the surface and can lead to heat accumulation in laser processes with a high repetition rate. Consequently, ablation by the next pulse starts at an increased surface temperature and affects the ablation threshold, which is consistent with the findings of this study.

In summary, scanning strategies should aim at high pulse overlap if fast modification of the surface, a maximized volume of ablated material and a high roughness increase is desired. An appropriate or relatively higher scanning line overlap leads to a homogenous distribution of nanostructures and helps avoid strong waviness of the laser treated surface. Several studies have already shown that laser induced periodic surface structures (FLIPSS, LIPSS) provide favorable conditions for cell attachment [15,17,58,59]. The impact of two selected micro and two selected FLIPSS structures presented in the current study on the cell adhesion of MG-63 cells have already been investigated [15]. It could be demonstrated, that the osteoblasts adhered tightly, especially to the two investigated FLIPSS-structured surfaces, and it was found that the spreading was superior on these surfaces. The findings of this cell study can be used in conjunction with the current study for tailoring of Ti6Al4V implant surfaces with improved cell behavior

It is reported in the literature, fs laser treatment affects the crystallographic, chemical, and mechanical properties of the sublayer on the surface in a range of several hundreds of nanometers [60,61,62] and, consequently, the implant performance. Therefore, further research should focus on the effect of laser treatment parameters on the dimension and properties of the heat affected zone (HAZ) and internal structure in the nano- and microstructures on Ti6Al4V, such as layer thickness, grain size and growth within the layer, and derived mechanical properties. Furthermore, to enhance the mechanical properties of the surface such as the tribological behavior or nanohardness, additional coating and sputtering processes [63,64,65] or chemical modifications of the titanium alloy or surface could be beneficial [66,67,68].

## 4. Conclusions

The effects of laser pulse overlap (*PO*) and scanning line overlap (*LO*), as well as applied fluence on the surface topography of Ti6Al4V, have been investigated in detail. Four different surface patterns could be identified depending on the applied laser parameters and scanning strategy. Micrometric ripples (MR) and femtosecond laser-induced periodic surface structures (FLIPSS) could be distinguished. Furthermore, the formation of micro-craters and pillared microstructures have also been observed. The microstructure is partially superimposed by the nanostructure, leading to a double scale formation. The average area surface roughness has been determined to quantify the ablation amount in order to specify the ablation regime. In particular, it has been found that the effects of *PO* and *LO* on the strong ablation regime are essentially different.

The results can be summarized as follows:

- It has been demonstrated that the strong ablation threshold depends on *PO* and *LO* as well as on the fluence.
- High *POs* cause extended phase explosion and heat accumulation which ultimately leads to a decrease of the strong ablation threshold.
- Higher *POs* should be preferred if high efficiency regarding the ablation amount and roughness is desired.
- A higher *LO* leads to a homogenous formation of nanostructures at low fluences with less waviness.

The influence of the pulse duration and the laser wavelength on the formation of nano and microstructures should be quantified in further studies to enhance laser processing strategies on Ti6Al4V, particularly with regard to high repetition laser processes and ultrafast laser systems. Lastly, the laser parameter findings presented in this study can be used for tailoring of proper structural designs of biomedical implants in order to adjust the cell material interaction of medical implants for dental and orthopedic applications. Further studies should also focus on the adhesion and proliferation of cells seeded onto laser-structured surfaces with regard to specific applications.

## Figures and Tables

**Table 1:** Laser parameter setting. Laser pulse and scanning line overlap were varied for each pulse energy/fluence value.

Laser Parameter Variations															
**Pulse energy (µJ)**	5	10		15		20		25	50		75		100		125
**Fluence (J/cm²)**	0.49	0.98		1.47		1.96		2.46	4.91		7.37		9.82		12.28
**Laser pulse overlap (*PO*) (%) at fix *LO* of 50%**	40		50		60		70			80		90			
**Scanning line overlap (*LO*) (%) at fix *PO* of 50%**	40		50		60		70			80		90			
